# Genomic Signatures of Domestication Selection in the Australasian Snapper (*Chrysophrys auratus*)

**DOI:** 10.3390/genes12111737

**Published:** 2021-10-29

**Authors:** Jean-Paul Baesjou, Maren Wellenreuther

**Affiliations:** 1 The New Zealand Institute for Plant and Food Research Ltd., 1025 Auckland, New Zealand; baesjoujeanpaul@gmail.com; 2 The New Zealand Institute for Plant and Food Research Ltd., 7010 Nelson, New Zealand; 3 School of Biological Sciences, University of Auckland, 1010 Auckland, New Zealand

**Keywords:** aquaculture, selective breeding, outlier scans, genome scans, selective sweep

## Abstract

Domestication of teleost fish is a recent development, and in most cases started less than 50 years ago. Shedding light on the genomic changes in key economic traits during the domestication process can provide crucial insights into the evolutionary processes involved and help inform selective breeding programmes. Here we report on the recent domestication of a native marine teleost species in New Zealand, the Australasian snapper (*Chrysophrys auratus*). Specifically, we use genome-wide data from a three-generation pedigree of this species to uncover genetic signatures of domestication selection for growth. Genotyping-By-Sequencing (GBS) was used to generate genome-wide SNP data from a three-generation pedigree to calculate generation-wide averages of F_ST_ between every generation pair. The level of differentiation between generations was further investigated using ADMIXTURE analysis and Principal Component Analysis (PCA). After that, genome scans using Bayescan, LFMM and XP-EHH were applied to identify SNP variants under putative selection following selection for growth. Finally, genes near candidate SNP variants were annotated to gain functional insights. Analysis showed that between generations F_ST_ values slightly increased as generational time increased. The extent of these changes was small, and both ADMIXTURE analysis and PCA were unable to form clear clusters. Genome scans revealed a number of SNP outliers, indicative of selection, of which a small number overlapped across analyses methods and populations. Genes of interest within proximity of putative selective SNPs were related to biological functions, and revealed an association with growth, immunity, neural development and behaviour, and tumour repression. Even though few genes overlapped between outlier SNP methods, gene functionalities showed greater overlap between methods. While the genetic changes observed were small in most cases, a number of outlier SNPs could be identified, of which some were found by more than one method. Multiple outlier SNPs appeared to be predominately linked to gene functionalities that modulate growth and survival. Ultimately, the results help to shed light on the genomic changes occurring during the early stages of domestication selection in teleost fish species such as snapper, and will provide useful candidates for the ongoing selective breeding in the future of this and related species.

## 1. Introduction

The breeding of plants and animals is one of the major transitions in history and its study can shed light into the genomic changes involved in the domestication process [1]. Changes to the genetic composition of a captive population can be introduced in a number of different ways. The first of these is through the relaxation of natural selection in the artificial environment [2]. Living in a controlled environment means species no longer face natural predators, have easy access to food and can be treated for diseases. Genotypes which are disadvantaged in nature may therefore be able to survive better in a more controlled environment that is devoid of food limitation and predators [3,4]. The second way is through the intentional human selection of economically important traits, as well as the introduction of unintentional novel natural selection caused by the new domestic environment [3,4]. Detecting the genetic signatures of neutral and selective processes in domestic populations provides information on the molecular processes that shape the genome following human-induced selection, as well as providing information for improving selective breeding programmes more generally.

Intentional selection for beneficial traits is expected to cause selective sweeps, increasing the frequency of the selected beneficial variants in that region of the genome [5]. A distinction is made between hard selective sweeps, where a beneficial variant sweeps through the population and becomes fixed, and soft selective sweeps, where multiple independent variants in the same locus sweep through the population simultaneously [6,7]. Revealing these variants and how they are involved with traits that are being selected for can give insight into the effectiveness of the breeding programme. A decade ago, the high acquisition cost of population-wide genomic data for a breeding populations was commonly prohibitive in performing population genomics studies for new aquaculture species. In recent years, however, the development of techniques such as reduced representation libraries (e.g., GBS, RAD) and genome-wide SNP arrays has allowed large amounts of genomic data to be gathered at an ever decreasing cost [8]. This has allowed for a range of increasingly accurate methods scanning the genome for signs of selection to be applied on a wider range of species than was previously possible [9].

Aquaculture in New Zealand is a rapidly expanding industry, with an annual revenue of about $NZ650 million in 2020. While New Zealand has long coastlines and one of the largest EEZs in the world, only three species are commercially farmed: Greenshell mussels (*Perna canaliculus*), Chinook salmon (*Oncorhynchus tschawytscha*) and Pacific oyster (*Magallana gigas,* formerly known as *Crassostrea*) [10]. To diversify the New Zealand aquaculture sector, the domestication of new aquaculture species is an important factor for economic growth and to ensure future resilience. The Australasian snapper *Chrysophrys auratus*, referred to as tāmure by the indigenous people of New Zealand (Māori), are a marine teleost of the family Sparidae, which can be found in the coastal waters of Australia, including Tasmania, and New Zealand. Snapper are of significant commercial, recreational and cultural importance, and a selective breeding programme was started in New Zealand in 2016 [11,12,13]. The aim of the current study is to uncover the genomic footprints of domestication selection in snapper following selection for increased growth performance and survival on a land-based facility. Three complementary genome scan methods were applied to a genome-wide SNP data derived through GBS of a three generation snapper pedigree: The F_ST_ outlier test Bayescan, the environmental association analysis LFMM and extended haplotype homozygosity analysis as applied in XP-EHH. We will present the findings from each analysis and annotate genes close to the regions of putative outlier SNPs. We then discuss our findings in light of domestication selection in this and other species. The new insights from this study will complement the ongoing research of the breeding success in this species, provide information on the early stages and targets of domestication, as well as provide target candidate genes for future research on economically important traits in this and related species.

## 2. Materials and Methods

### 2.1. Study Populations: Pedigree Structure and Information

The snapper populations in this study were obtained as part of a finfish breeding programme at the Nelson Finfish Facility of Plant and Food Research in New Zealand and consisted of a wild-caught F_0_ generation, with an F_1_ and F_2_ generation spawned in captivity. The F_0_ generation consisted of two cohorts of 25 individuals. The first cohort was caught from several sites around the Tasman Bay, New Zealand in 1996. The second cohort was caught from a single site within the Tasman Bay, New Zealand in 2006. Previous genetic studies [14] as well as a recent genomic investigation of wild snapper populations [15] (Wellenreuther unpublished data) show that genetic diversity of wild snapper is very homogenous across New Zealand, and similar to what has been found in other fish with large population sizes including Atlantic cod (*Gadus morhua*) [16], blue whiting (*Micromesistius australis*) [17], and herring (*Clupea harengus L.*) [18], indicating that these samples have captured the genetic diversity of snapper in the Tasman bay area, and can be also seen as a roughly representative of the expected amount of genetic diversity of snapper stocks in New Zealand.

Over time, an F_1_ generation of individuals was produced from subsequent spawning events of individuals derived from the 1996 and 2006 cohorts of the F_0_ generation. Individuals of the F_1_ generation were eventually combined into a single F1 broodstock and used to produce an F_2_ generation [12,13]. Spawning of broodstock fish was achieved using mass spawning, which is the typical method for most bream species, with equal sex ratios and all individuals able to mate freely with other individuals in the population. Prior to the spawning broodstock fish were fed a specialized diet containing fresh fish and oil supplements. Fertilized eggs were collected from the tank outlet during over consecutive days. All cohorts were subjected to domestication selection for improved growth and survival. Growth in snapper has been assessed in detail as part of this selective breeding programme [11,12,13,19,20], and the work has shown that growth traits (e.g., length or weight) have a strong positive allometric relationships and can this be used as good proxies for one another. The growth selection applies an image based method using the software package Morphometric Software™ (https://www.plantandfood.co.nz/page/morphometric-software-home/, accessed on 14 September 2021). The software extracts the outline of each individual fish from images, locates the XY coordinates of morphometric features on the outline (e.g., upper lip and narrowest cross section of the tail) and then uses those coordinates to make measurements. The measurements were converted from pixels to mm using the length of rulers also present in the images. Breeding selection for growth has been based on a combination of length and weight traits.

### 2.2. Sampling and Generation of Molecular Data

Sampling and generation of SNP data were performed in an earlier study [12,13]. In short, samples of finclip tissues were collected for all fish, which included part of the second cohort of the F_0_ generation as well as the full F_1_ and F_2_ generations. The first cohort of the F_0_ generation had by this time already died and no samples could be taken.

Genome-wide SNP data were generated through the Genotyping-By-Sequencing (GBS) method [21]. GBS makes use of restriction enzymes to cut the DNA at conserved regions across the genome and then sequencing the associated DNA at that region, thereby reducing the overall representation of genomic data. The resulting fragments are then sequenced using next-generation sequencing (typically using an Illumina machine, San Diego, CA, USA) [21]. Libraries were double digested using the restriction enzymes *Pst*I and *Msp*I and barcode adapters were annealed to distinguish reads belonging to different individuals for downstream analyses. The libraries were amplified separately before being prepared in parallel plates. Duplicate or triplicate samples were prepared for the F_0_ and F_1_ generation, as well as two individuals of the F_2_. The plates were pooled and then sequenced using Illumina HiSeq 2500 platform in single-end (SE) mode, with a read length of 100 bases. This resulted in eight FASTQ files being produced, one for each sequencing lane. Also generated from earlier research were a snapper reference genome and associated GFF3 gene annotation file [15].

### 2.3. Raw Reads, Mapping, Variant Calling, and Filtering of the Data

An overview of the data processing pipeline is given in Supplementary Figure S1. Raw GBS FASTQ files were demultiplexed using the process_radtags module which is part of the STACKS v2.2 pipeline [22,23]. During demultiplexing, sequence data were collectively gathered in a single FASTQ file which was then split into sample-specific FASTQ files using DNA barcodes. From demultiplexing, eight barcode files were generated, one for each sequencing lane. After demultiplexing, duplicate and triplicate sample FASTQ files were concatenated. Adapters were clipped and raw reads were trimmed using cutadept v1.15 [24]. A PHRED quality score cut-off of 33 was used, to ensure only high-quality SNPs would be used in subsequent analyses. A minimum read length of 50 was specified, to ensure reads could be reliably mapped. The adapter sequence provided to cutadept consisted of only the first 13 nucleotides of the full adapter. This was done because the full adapter sequence is unlikely to be found within the read. The trimmed FASTQ files were aligned to the reference genome using BWA-MEM v0.7.17 [25]. Before starting a run of BWA, an Index database of the reference was generated using bwa index. Sorted BAM files were generated from BWA output using the view and sort commands from Samtools v1.9 [26]. Variant calling was performed in two steps using the gstacks and populations modules from STACKS v2.2; settings included a population file with -M and --write_single_snp. This resulted in four VCF files being generated. One for each pair of generations and one that included all generations. In order to remove variants that were either uninformative or likely to cause false positives in downstream analyses, all VCF files were filtered using VCFTools v0.1.14 [27]. Variants with a low read depth across individuals run might not include all alleles, which can lead to inaccurate estimations of allele frequency. This can then result in inaccurate estimation of the F_ST_, as well as causing false positives/false negatives in F_ST_ outlier tests [28]. Thus, sites with an average depth of less than 10 across individuals were removed using the min-meanDP flag. In addition, variants that are only captured in few individuals cannot be used in most analysis without requiring imputation. Because of this, variants that were genotyped in less than 90 percent of individuals were removed using the max-missing flag. Variants with a minor allele frequency of 0.05 or less were removed using the max flag. These rare variants are generally considered to lack the sensitivity required to show signatures of drift and hitchhiking, making them uninformative in genome scan methods that rely on allele frequencies and should be removed before performing such analyses [29]. Finally, the data were filtered to only contain biallelic SNPs using the min-alleles and max-alleles flags.

### 2.4. Analysis of Genetic Differentiation between Generations

The expected and observed heterozygosity H_E_ and H_O_ were calculated using diveRsity [30], as well as F_IS_ and associated 95% confidence intervals. The number of bootstrap replicates was set to 100. To investigate the level of genetic differentiation between cohorts, Weir and Cockerham’s F_ST_ [31] estimate was calculated with 95% confidence intervals for all possible generation pairs using diveRsity. The number of bootstrap replicates used when calculating confidence intervals was set to 100 and bootstrapping was carried out over individuals within samples.

### 2.5. ADMIXTURE Analysis

Genetic ancestry was estimated through ADMIXTURE v1.3 [32]. Input files were generated using Plink [30]. The program uses an unsupervised approach to calculate a matrix of ancestry coefficients, which are proportions of an individual genome estimated to belong to different ancestral populations. Admixture requires the user to supply the expected number of ancestral populations K. Runs were performed using values of *K* between 1 and 3.

### 2.6. Principal Component Analysis

Adegenet v2.1.3 was used to perform PCA on matrices of allele frequency [33]. PGDSpider v2.1.1.5 was used with a population file to transform the VCF file containing all individuals to the genepop format [34]. In the SPID settings file, the population definition file parser question was set to yes, while all other VCF parser questions were left at default values. For the genepop writer questions, the datatype was set to SNP data, while other writer questions were left at the default values. The genepop file was then stripped of headers and transformed into a genind object using the read.table and df2genind functions. PCA was performed using the dudi.pca function.

### 2.7. Identifying SNP Variants Associated with Selective Sweeps

Bayescan v2.1 is an F_ST_ outlier method based around the multinomial-Dirichlet distribution [35]. This software works by first decomposing locus-population F_ST_ coefficients into a locus-specific component and a population-specific component. It then creates two different models for every locus, one of which includes the locus specific component and one that does not. The posterior probabilities for both models are then calculated using a reversible-jump MCMC approach. The use of posterior probabilities combined with setting prior odds for the model without selection allows direct control of the FDR. This allows for the defining of *q*-values which can in turn be used to make decisions on outlier loci [35]. PGDSpider v2.1.1.5 was used to reformat VCF files for each comparison to the Bayescan format. VCF parser settings were left at their defaults, while the datatype for the GESTE/Bayescan writer questions was set to SNP data. Bayescan was then run for all comparisons using the default settings.

LFMM is an EAA method used to detect SNP variants that were potentially being affected by positive selection as a result of domestication while accounting for background population structure [36]. LFMM attempts find correlations between allele frequency and an environmental factor using linear associations, which in our case was based on different comparisons between the F_0_, the F_1_ and the F_2_ cohorts. Latent factors are used to correct for background population structure. The significance of the correlation is shown through a *p*-value for each SNP variant [37,38]. LFMM has been implemented as part of the lea R package v2.8.0, along with SNMF, a tool for inference of ancestry coefficients, as well as various other utility functions [39]. Environment files for each VCF file were generated, and individuals from different cohorts were separated by environmental value. Individuals from the F_0_ cohort were given the value of 0, those of the F_1_ cohort received the value of 0.5 and those of the F_2_ cohort received the value of 1.The “lfmm.R” script was then used to run SNMF impute missing values and run LFMM. SNMF was set to run for 5 repetitions, entropy turned on and was set to override previous results. The best SNMF result was then used to impute the LFMM input data. LFMM was then run with 10,000 iterations and 5000 burn-in iterations. To determine the correct number of latent factors for use with SNMF and LFMM, runs were completed using *K* = 1 and *K* = 2. The histograms of *p*-values were then compared for different values of K. Finally, output *p*-values were adjusted for multiple testing using the method as implemented in the R package qvalue [40].

XP-EHH analysis was performed using the R package rehh [41,42]. Rehh was developed to enable application of EHH on large genome-wide datasets to uncover footprints of selection. XP-EHH attempts to find variants under selection by looking for those variants with extended haplotypes where drift has not deteriorated the frequency of variants linked to the variant under selection. It does this by comparing the integrated EHH profiles for the same Variant between two different populations. Significance for this comparison is shown through a *p*-value for each SNP variant. The program accepts haplotype VCF input directly and can efficiently calculate a wide variety of EHH statistics [42]. Three VCF files containing individuals of the F_0_, F_1_ and F_2_ respectively were first sorted by chromosome and position using bash and then phased and imputed using BEAGLE v5.1 in order to produce haplotypes required for rehh. The haplotype VCF files were then loaded into R using the data data2haplohh function. Polarization was set to “False” to indicate that no outgroup genome was used to set alleles as derived or ancestral. Next the iES statistic, the site specific integrated EHH, was calculated for every site in each population using the scan_hh function. Polarization was once more set to “False” Finally, XP-EHH was calculated for each pair of populations using the ies2xpehh function. Derived *p*-values were corrected using the method of Benjamini and Hochberg implemented in the ies2xpehh function [43].

Significance cut-offs for each method were applied as follows. For Bayescan, we retained outlier SNPs with a *q*-value ≤ 0.05 (leading to a FDR of ≤0.05). The *q*-value is the false discovery rate (FDR) analog of a *p*-value; it is the minimum FDR at which a locus may become significant. A *q*-value of 0.05 means that 5% of outliers (i.e., those having a *q*-value  ≤ 0.05) are expected to be falsely positive. A 5% threshold for *q*-values is much more stringent than a 5% threshold for *p*-values in classical statistics. For LFMM, a *q*-value threshold of 0.05 was used. Finally, a *q*-value threshold of 0.05 was also used for XP-EHH. Venn diagrams showing overlapping outliers were made using the R package eulerr v6.1.0 [44]. Three VCF files were created, which contained the putative loci detected by all methods for each comparison of generations using positional data on each variant with VCFtools –positions [27].

### 2.8. Annotation of Genes near Putative Variants

Three VCF files were created, which contained the putative loci detected by all methods for the comparisons of each generation pair using positional data on each variant with VCFtools –positions. The VCF files were then transformed to the BED and a bedtools genome file, containing the sizes of each chromosome, was generated from the reference genome’s index file [45,46]. Bedtools slop was then used to increase the feature size of the variant data by 10 kb in both directions. This essentially decreases the starting position by 10 kb and increases the end position by 10 kb. After that, bedtools intersect was used to find features on the gff3 annotation file which overlapped with the extended variant features. The –wb flag was used to output only the entries from the GFF3 file that overlapped with the variant features. A new GFF3 file was then created from the output. Finally, bedtools –getfasta was used to generate a fasta file which contains every feature on the gff3 file that intersected with one of the putative variants. Bedtools –getfasta takes a file in the GFF3 format, along with a FASTA genome as input. It uses the positional data in the GFF3 file to extract sequences from the FASTA genome and creates a new FASTA file with one line in the FASTA output for every line in the input file. To our knowledge, no high-quality protein database exists for snapper as of writing, so the zebrafish (*Danio rerio*) protein database from ensemble was used instead. The blast protein database was then constructed using the makeblastdb command, with dbtype set to “prot”. The blastx command was used to find regions of similarity between translated nucleotide sequences from features on the GFF3 file and the zebrafish protein database. The blast search was limited to only show one result for each feature. An e-value threshold of 1 × 10^−3^ was maintained. The ensemble versioned protein identifiers of proteins that were linked to features of interest were used in a biomart query to find the associated ensemble gene name.

## 3. Results

### 3.1. Genotyping and Quality Control

The provided starting data consisted of one reference genome, along with 6,515,701,876 reads for 662 individuals. After applying filters to remove low-quality reads 5,545,018,856 reads remained for further analysis. Using the cleaned reads, one set of SNPs was generated for each of the three comparisons. The subset containing individuals from F_0_ and the F_1_ populations produced 226,618 SNPs, the subset containing individuals from the F_1_ and the F_2_ populations produced 254,342 SNPs and the subset containing individuals from the F_0_ and the F_2_ populations produced 254,299 SNPs. Filters for average read depth, genotype call rate, minor allele frequency and biallelic alleles were applied to every subset. After filtering, the final subsets for F_0_ and F_1_ contained 33,264 SNPs, the subset for F_1_ and F_2_ contained 17,262 SNPs and the subset for F_0_ and F_2_ contained 14,629 SNPs.

### 3.2. Analysis of Genetic Differentiation between Generations

Based on the filtered set of SNPs, the populations were compared to gain insights into the extent of genetic differentiation (Table 1A).Values of the expected heterozygosity (H_E_), the observed heterozygosity (H_O_) and the inbreeding coefficient were largely similar, but also showed an overall small increase in differentiation as domestication time increased (confidence intervals did not overlap between F_0_ and the F_2_ populations, indicating significance at *p* < 0.05, see Table 1). This was also reflected in the F_ST_ values between population pairs. Table 1B shows the F_ST_ values for the different pairs of populations, with a higher F_ST_ showing a larger proportion of the total genetic variation between populations compared to within populations. The results show the F_ST_ between the F_0_ and the F_1_ generation is smaller than the F_ST_ between the F_1_ and the F_2_ generation (significant comparisons at *p* < 0.05 are depicted in bold, for all comparisons). The greatest level of differentiation is found between the F_0_ and the F_2_ populations, again indicating a slight increase in genetic differentiation as time of since domestication increases.

### 3.3. ADMIXTURE Analysis

**Figure 1:**
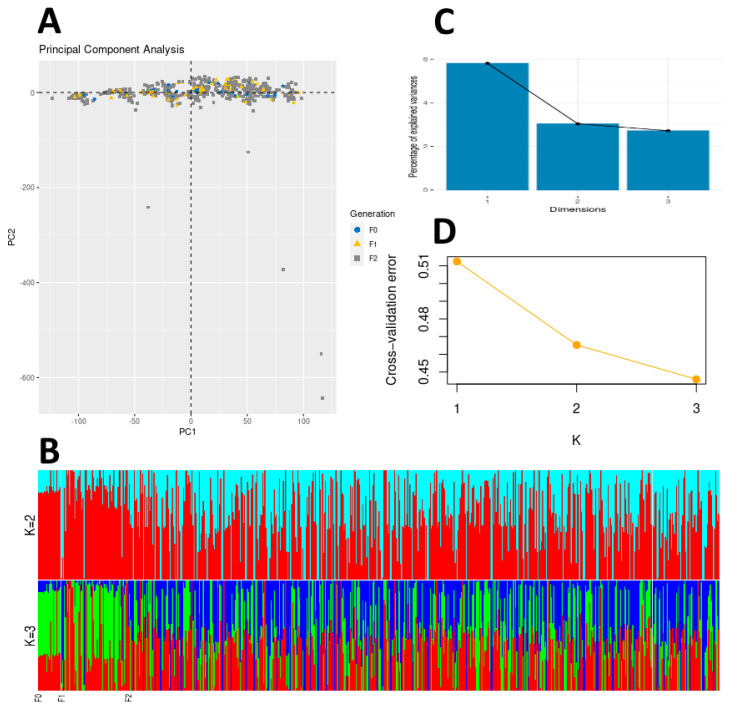
(**A**) PCA plot generated from Adegenet analysis. The principal components represent different sources of genetic variance, and points on the graph represent individuals, while colours and shapes of each point represent different generations. If distinct groups exist within the sampled individuals, then they will form different clusters. (**B**) Barplot of ADMIXTURE analysis results for *K* = 2 and *K* = 3. ADMIXTURE analysis calculates ancestry coefficients, which is the proportion of an individual genome that belongs to a certain ancestral population. The x-axis contains all individuals, grouped by generation from F_0_ to F_2_. The y-axis shows, in different colours, the proportions of the individuals’ genome belonging to different ancestral populations. *K* is the number of ancestral populations used in the analysis. (**C**) Scree plot showing the percentage of variance explained by the first three principal components from Adegenet PCA. (**D**) Cross-validation error for different values of *K* generated by ADMIXTURE.

ADMIXTURE analyses was used infer the origins based on genetic ancestry (Figure 1). The matrix of ancestry coefficients generated by ADMIXTURE was plotted for *K* = 2 and *K* = 3 (Figure 1B). The ADMIXTURE results do not show strong population differences as seen by the lack of clear distinct groupings, although a change in the assigned populations is visible between the F_0_/F_1_ and the F_2_ generations. This can be seen by the increase in the blue ancestry coefficient colour. Analysis of the cross-validation error for different values of *K* is shown in Figure 1D and reveals that *K* = 3 produces the lowest cross-validation error, indicating that the likely number of genetic clusters is 3.

### 3.4. Principal Component Analysis

The principal components 1 and 2 derived by Adegenet PCA were plotted in order to explore genetic clusters in the data. The results are shown in Figure 1A. As the principal components represent different sources of genetic variance, individuals should form clusters if genetically distinct groups are present. Like the ADMIXTURE analysis however, the PCA did not reveal genetic clusters associated with specific snapper breeding populations. However, while the PC1 capture the vast majority of variation of the data, some individuals from the F_2_ population were spread along the PC2 axis. This spread of F_2_ individuals indicates that genetically different variance in this cohort is the greatest, suggestive of stronger and sustained selection in this generation.

### 3.5. Identifying SNP Variants Associated with Selective Sweeps

**Figure 2:**
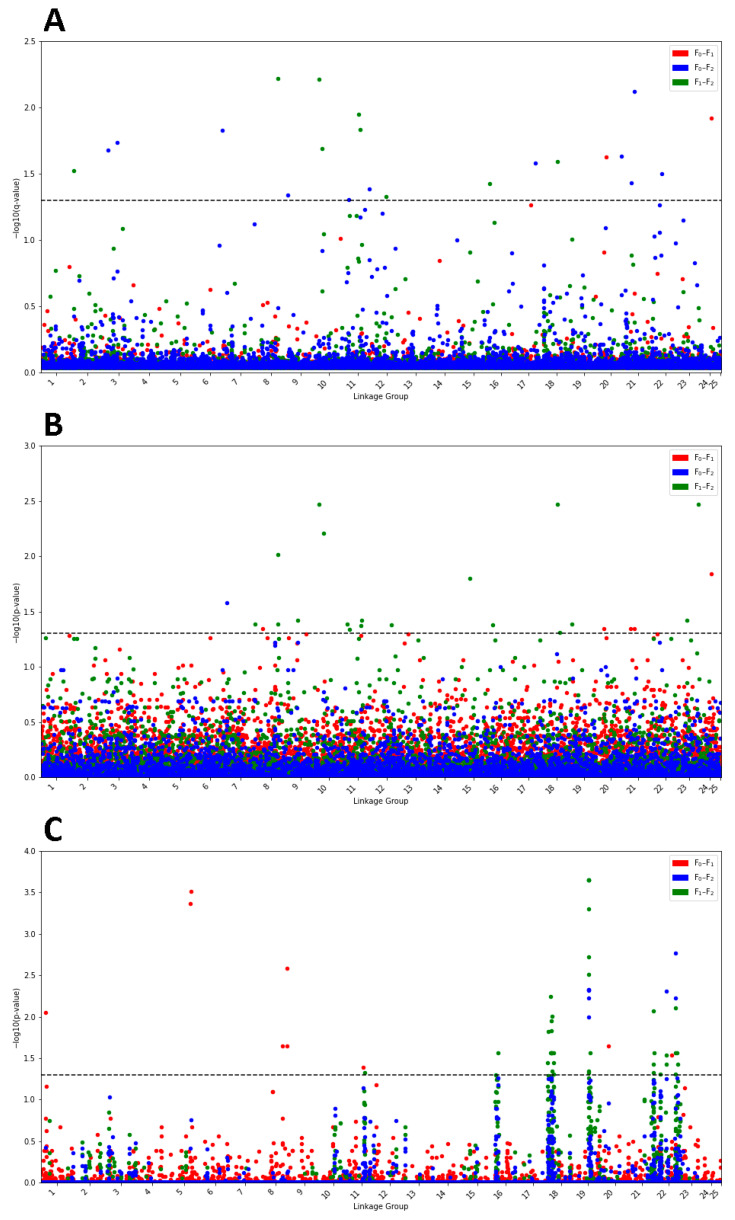
(**A**) Manhattan plot of Bayescan analysis for every locus for every pair of populations. The x-axis displays the linkage group a locus is located. Position on the x-axis indicates the variants’ position in the linkage group. The y-axis shows the −log10 *q*-value attached to the locus. The dashed line indicates the significance threshold. (**B**) Manhattan plot of LFMM analysis for every locus for every pair of populations. The x-axis displays the linkage group a locus is located. Position on the x-axis indicates the locus’ position in the linkage group. The y axis shows the −log10 *q*-value attached to the locus. The dashed line indicates the significance threshold. (**C**) Manhattan plot of XP-EHH analysis for every locus for every pair of populations. The x-axis displays the linkage group a locus is located. Position on the x-axis indicates the locus’ position in the linkage group. The y axis shows the −log10 *q*-value attached to the locus. The dashed line indicates the significance threshold.

**Figure 3:**
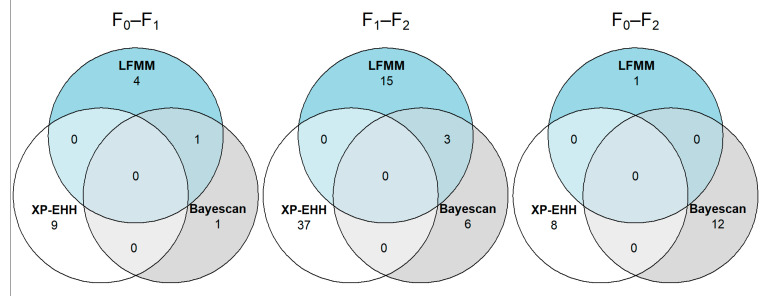
Venn diagrams showing overlapping loci considered significant between different methods for every pair of populations.

Bayescan was used to detect SNP variants potentially affected by positive selection as a result of domestication. Figure 2A shows the Manhattan plot containing the −log10 *q*-values for every SNP variant between every pair of cohorts, as well as the linkage group it is located in. Variants were considered to be significant for *q*-values < 0.05. Bayescan revealed 2 significant SNP variants between the F_0_ and the F_1_ cohorts, 9 between the F_1_ and the F_2_ cohorts and 12 between the F_0_ and the F_2_ cohorts. LFMM implemented in the R package lea was used to detect SNP variants that were potentially being affected by positive selection as a result of domestication. Supplementary Figure S2 shows the histograms of *p*-values generated for each comparison between cohorts with the number of latent factors *K* set to 1 and 2 (Figure 2B). Variants were considered to be significant for *q*-values < 0.05. LFMM revealed 5 significant SNP variants between the F_0_ and the F_1_ cohorts, 18 between the F_1_ and the F_2_ cohorts and 1 between the F_0_ and the F_2_ cohorts. XP-EHH implemented in the R package rehh was used to detect SNP variants that were potentially being affected by positive selection as a result of domestication (Figure 2C). Variants were considered to be significant for *q*-values < 0.05. XP-EHH revealed 9 significant SNP variants between the F_0_ and the F_1_ cohorts, 37 between the F_1_ and the F_2_ cohorts and 8 between the F_0_ and the F_2_ cohorts. Figure 3 shows a Venn diagram of all of the overlapping putative variants between the three methods: Bayescan, LFMM and XP-EHH.

### 3.6. Annotation of Genes near Putative Variants

BLAST revealed 17 unique protein-coding genes in proximity to SNP variants found to be putative between the F_0_ and F_1_ cohorts, 96 between the F_1_ and F_2_ and 24 between the F_0_ and F_2_ cohorts (Table 2). These genes, along with the SNP variant position on the chromosome and the methods that identified the SNP variant as putative, can be found in Supplementary Tables S1–S3. Of these genes there were only 10 that overlapped between comparisons and all of these overlapped between the comparison between the F_1_ and the F_2_ and the comparison between the F_0_ and the F_2_. In addition, all of the overlapping genes were found near SNPs discovered by XP-EHH analysis. Overlapping genes are listed in bold in Supplementary Tables S1–S3.

## 4. Discussion

Investigating genetic changes over time in domesticated populations can provide insights into the regions of the genome under selection [47]. In this study, the genetic signatures of selection in a three-generation pedigree of the Australian snapper (*C. auratus*), selected for improved growth and increased survival, were investigated. The results showed that overall, genetic differentiation between generations was weak yet very slightly increasing over generational time. Furthermore, we were able to identify for the first time putative genes involved in the domestication selection of this new species for aquaculture. We compare our results to other studies and highlight wider implications and next steps.

The large SNP dataset for the three-generation snapper pedigree was first used to explore the genomic characteristics of the three generations in more detail with the aim to provide some context about the genetic make-up of the populations. We achieved this by calculating the pairwise F_ST_ values between all three cohorts (F_0_ vs. F_1_, F_0_ vs. F_2_ and F_1_ vs. F_2_) and found that all pairwise F_ST_ values were low yet significant between both the F_0_ vs. F_2_ and F_1_ vs. F_2_. It should be noted that even though these significant differences were very weak, we detected that the level of differentiation increased subtly with generational time, as indicated by the increased F_ST_ between the F_0_ and F_2_ cohorts compared to the values between all other cohorts (Table 1). This indicates that genetic differentiation slightly increased over time, consistent with ongoing domestication selection. These results were consistent with the findings of the ADMIXTURE analysis and the PCA, which both again detected an overall low level of differentiation that was most pronounced in the latest F_2_ generation, consistent with sustained and ongoing selection in this species.

We then investigated the genome SNP dataset to identify regions that are under putative domestication selection. For this, we applied three different genome scan methods because we are aware that each method has its own limitations and strengths and we wanted to explore if we can identify SNPs or genes that are found by more than one method [48]. Our *a priori* expectation is also that selection signatures would be very weak at this stage, as this study details only the very first changes of genome evolution following selective breeding, which is the main point of interest and novelty of this study. Gaining insights into what may allow one species to survive and become adapted to a new artificial environment (e.g., a land based finfish facility), and in addition, perform well with regards to growth and survival, has the potential to uncover important insights into what makes species resilient and amendable to domestication.

We found that SNP outlier overlap between the three methods was low (Figure 3), and the only genes of interest overlapping between different comparisons were *map2k4a* and *map2k4b* (Supplementary Tables S2 and S3).

The lack of overlap between outliers produced by different methods is likely caused by the differences in the underlying mechanisms behind each method. Because of these differences, it is logical for each method to possibly return a different group of outliers, all of which can still be considered as valid putative outliers. In short, Bayescan is an F_ST_ outlier detection method which is mostly going to detect strong signatures of selection associated with fixated variants. F_ST_ outlier detection methods are prone to detect many false positives if the population history is different from that which is assumed in their null models. One of these assumptions is that both populations evolved independently from a common ancestor. Since this is not the case for the populations in this study, this leaves potential for further false positives [49]. A final source of false positives might come from the use of a fairly lenient posterior odds value of 10, indicating the neutral model is 10 times more likely to occur than the selection model. While this value can be considered low for the number of loci, this value has been used in other publications with similarly sized datasets [50]. LFMM is an EAA method based around linear associations, which is better at detecting more subtle signatures of selection associated with soft sweeps. In addition, LFMM can better account for complex population history and correct for neutral population structure. In the case of this study, the F_1_ and F_2_ cohorts were formed from two separate F_0_ cohorts, which lead to the chosen number of latent factors used being 2. There is some level of uncertainty involved with this, as the two ancestral populations were caught from the same site and genomic work suggest significant genetic homogeneity of snapper from Tasman Bay, and little generational time had passed between the two catches. This could mean the populations are not genetically distinct enough for *K* to be 2, rather that would mean *K* could be 1. Comparing the histograms for runs using *K* = 1 and *K* = 2 reveals the histograms are extremely similar, supporting the claim that F0 cohorts are genetically identical. The histograms of a properly calibrated LFMM run are expected to mostly be flat, with a peak near minimal *p*-values [39]. Because of this, it is unlikely that this difference would cause a dramatic shift in results, but significance values could shift slightly as a result. XP-EHH is a method based on the comparison of haplotypes, which means it will be mostly detect recent signs of selection. This because is extended haplotypes inevitably fade over time due to genetic drift reducing the frequency of variants that were closely linked to a variant under selection and only increased in frequency due to the effect of hitchhiking. It is limited by the accuracy of the haplotypes supplied to it. When producing haplotypes using BEAGLE, there was no access to a plink map file which caused BEAGLE to assume a constant recombination rate of 10 cM per Mb. This may have led to some inaccuracies when calling haplotypes, causing false positives in the analysis. XP-EHH was the only method to find outliers that overlapped between comparisons of cohorts. In addition to differences in the methods, differences in cohort sizes could also have had various effects on the results of each method. In F_ST_ outlier detection tests, the power to reject neutrality is maximized when sample sizes of the groups in the comparison are close to being even. In environmental association tests, power is maximized when samples are spread out over a large geographic area and not clustered into groups. These properties can cause uneven sampling designs to have reduced power [51]. The impact of varying cohort sizes also goes beyond uneven sampling. It should be noted that the comparison with the highest number of individuals, the comparison between the F_1_ cohort and the F_2_ cohort also featured the most putative variants. It is therefore likely that a larger population size is beneficial when performing genome scans and that the small size of the F_0_ generation limits the power of genome scan methods. Finally, the small number of generations could have caused polygenic traits affected by selection to leave traces too subtle to reach the significance threshold of genome scan tests [52]. While genome scans have been used previously to discover signatures of domestication in Atlantic salmon by comparing wild with domesticated populations, it should be noted that the populations used in those studies were not direct offspring of one another and are therefore far easier to distinguish genetically [50,53].

Despite the general low overlap between methods, we found that once we annotated the genes to identify biological functions that the identified putatively selected genes in each comparison tended to impact similar biological functions. These functions were predominately confined to the categories: Growth, immunity, neural development and behaviour, and tumour repression (Table 2, Figure 2 and Figure 3). A recent study comparing signatures of domestication in two Atlantic salmon (*Salmon salar*) populations with different geographical origins reported similar results, with genes under selection theorized to have comparable and similar effects on growth, immune response and behaviour [54]. Below we detail the genes that were implicated to be under selection for each comparison, and briefly discuss what is known about their supposed function.

When comparing the F_0_ and the F_1_ cohorts, we identified five genes in close proximity to putative variants linked to potential domestication traits, i.e., traits associated with growth (Table 2). The *wars1* gene has been linked to body fat distribution in humans (*Homo sapiens*) and the *tbx15* gene has been linked to body size in goats (*Capra hircus)* and studies have found that it is essential to skeletal development [55,56]. We also identified the *spry2* gene as another candidate genes involved in domestication selection in snapper, which works as a feedback inhibitor to epidermal and fibroblast growth factors to stimulate cell growth in humans [57]. The two remaining genes that we identified were found to be related to neural development. The first of these is the *cntnap3* gene, which has a crucial role in the synaptic development and social behaviour in house mice (*Mus musculus*) [58]. The second is the *actr10* gene, which has a role in nervous system development and disease, and has been implemented in the domestication of red fox (*Vulpes vulpes)* populations with markedly different behavioural phenotypes [59]. Signatures of selection near these genes could be signs of behavioural changes, but further work on this would be needed to verify this.

The comparison between the F_1_ and the F_2_ cohorts showed 16 different genes of interest near putative SNP variants, the highest number of genes across all comparisons. Of these, four were found to be related to muscle growth. The first of these was the *cast* gene, which serves as an inhibitor of muscle protein degradation and is associated with muscle growth in Bali cattle (*Bos domesticus*) [60]. The second one is the *mef2b* gene which has been linked to regulation of muscle growth in sheep (*Ovis aries*) and is hence associated with general body growth and weight [61]. The final two are the *dock1* and *dock5* genes, which are both required for myoblast fusion during muscle development in zebrafish [62]. The next set of four genes was found to be related to neurodevelopment and behaviour. The first of these is the *epha6* gene which was found to be associated with temperament in Guzerat (*Bos indicus*), a Brazilian breed of domestic cattle [63]. The second one of these is the *fmn1* gen which appears to be associated with energy and trainability in dogs (*Canis familiaris*) [64]. Another one was the *dyrk1ab* gene, where knockout studies in zebrafish found it to be associated with traits related to social deficiencies relevant to autism [65]. Finally, the *p2ry1* gene, which may be connected to the eating habits of Chinese domestic pigs (*Sus domesticus*) [66]. As in the comparison between the F_0_ and the F_1_, this set of genes could present an early sign that behavioural changes are occurring as a result of domestication selection, for example, due to relaxed selection in the artificial farm environment. There were also a number of genes related to immune response. The first gene was *ephb2a*, which has been linked to immune and stress response in channel catfish (*Channel catfish*) [67] and the gene *ftr67*, which has been implicated to play an important role in the innate immune system of zebrafish [67]. Similarly, studies on the *irf2bp1* gene show that this gene has an important role in the immune system through macrophage regulation and lymphocyte activation in varied species [68] and likewise, the *arl16* gene has also been discovered to serve a function in the immune system in diverse species, including mammals [69]. Frequency changes in these genes could occur as a result of new diseases occurring and transmitting rapidly between individuals in the novel captive environment. Finally, there were several genes which had functions related to tumour suppression. For example, studies have shown that the *lxn1* gene is significantly downregulated in humans suffering from gastric carcinomas, marking it as a potential tumour suppression gene [70]. Other studies have shown that the *emilin2* gene causes apoptosis in a number of human tumour cells and also enhances tumour neo-angiogenesis [71]. Lastly, the *map2k4a* and *map2k4b* were implicated to be likely candidates for tumour suppression after missense mutations were associated with multiple carcinomas in humans [72].

The comparison between the F_0_ and the F_2_ cohorts yielded a further five candidate genes of interest. One of these genes, *rnpc3*, was found to be related to body size. The gene is part of a pathway which also include *igf1* and *igfr1*, both which have been shown to be related in body size in dogs [72]. As in the comparison between the F_0_ and the F_1_, this set of genes is likely to be under direct selection for growth. Four genes were related to tumour suppression: these included the *map2k4a* and *map2k4b* genes that were also detected in the F_1_ and the F_2_ cohort comparison, as well as the *ext1a* and *ext1b* genes, the latter which have also been revealed as putative tumour suppressors [72,73]. The final two genes have been found to be involved in neural development and disorders. Several variants of the first gene *zmz1*, were linked to intellectual disability and development delay in humans [74]. The second gene *mdga1*, was connected to schizophrenia in humans through association analysis and put forth as a new susceptibility gene [75]. As in earlier comparisons, this is in line with these genes having a neurodevelopmental role and being linked to behavioural changes as a result of domestication.

Taken together, our findings indicate that snapper is showing signs of increasing differentiation with ongoing and sustained selection in response to a new environment and selection for enhanced growth. Our study identified a first set of possible tentative candidate genes that are under selection as part of this process. Many of our identified genes point towards functions that appear to be related to growth, immunity and survival, but these links should only be seen as suggestive at this stage, as direct investigations of these genes in the study species, or even closely related teleost fish species, are absent to date. Future work on this and related species is needed to investigate the presence of a general pattern across species, and to verify or reject a role of the identified genes in the domestication process.

## 5. Conclusions

Domestication has left subtle but noticeable signatures of selection throughout the genome of the snapper populations studied in this work. This species has been selected as a new candidate species for aquaculture in New Zealand, and is of significant cultural, recreational and commercial value in this country. Genome scan methods have been used to designate a number of key genes associated with a set of putatively important biological functions of interest such as growth, immunity, neural development and behaviour, and tumour repression as likely targets of selection as a result of domestication. These findings serve as a first step to shedding light on the impact of domestication on the genome and serve as a stepping stone for future studies which seek to investigate in detail the impact of domestication on individual genes.

## Figures and Tables

**Table 1:** (A) Overview of population statistics. These include the number of individuals in the population (N), the expected heterozygosity (H_E_), the observed heterozygosity (H_O_) and the inbreeding coefficient (F_IS_) and the 95% confidence intervals (in brackets, with upper and lower confidence intervals being separated by a slash). (B) Overview of the average F_ST_ between pairs of populations (and the 95% confidence intervals in brackets, with upper and lower confidence intervals being separated by a slash). F_ST_ values in bold are significant.

A					B	
	N	H_E_	H_O_	F_IS_ (95% CI)		F_ST_ (95% CI)
F_0_	22	0.31	0.31	−0.024 (0.04/0.012)	F_0_–F_1_	0.0085 (0.0021/0.0211)
F_1_	65	0.32	0.33	−0.049 (0.062/0.035)	F_1_–F_2_	0.0214 (0.0201/0.0231)
F_2_	575	0.32	0.34	−0.064 (0.071/0.058)	F_0_–F_2_	0.0367 (0.0359/0.0376)

**Table 2:** Overview of the genes along with the function they are associated with, as well as the populations used in the comparison.

Gene Name	Comparison	Associated Function
*wars1*	F_0_–F_1_	growth/body shape/body size
*tbx15*	F_0_–F_1_	growth/body shape/body size
*spry2*	F_0_–F_1_	growth/body shape/body size
*cast*	F_1_–F_2_	growth/body shape/body size
*mef2b*	F_1_–F_2_	growth/body shape/body size
*dock1*	F_1_–F_2_	growth/body shape/body size
*dock5*	F_1_–F_2_	growth/body shape/body size
*rnpc3*	F_0_–F_2_	growth/body shape/body size
*cntnap3*	F_0_–F_1_	behaviour/nervous system
*epha6*	F_1_–F_2_	behaviour/nervous system
*fmn1*	F_1_–F_2_	behaviour/nervous system
*dyrk1ab*	F_1_–F_2_	behaviour/nervous system
*p2ry1*	F_1_–F_2_	behaviour/nervous system
*zmz1*	F_1_–F_2_	behaviour/nervous system
*mdga1*	F_0_–F_2_	behaviour/nervous system
*ephb2a*	F_1_–F_2_	immune response
*ftr67*	F_1_–F_2_	immune response
*irf2bp1*	F_1_–F_2_	immune response
*arl16*	F_1_–F_2_	immune response
*lxn1*	F_1_–F_2_	tumour suppression
*emilin2*	F_1_–F_2_	tumour suppression
*Map2k4a*	F_1_–F_2_, F_0_–F_2_	tumour suppression
*Map2k4b*	F_1_–F_2_, F_0_–F_2_	tumour suppression
*ext1a*	F_0_–F_2_	tumour suppression
*ext1b*	F_0_–F_2_	tumour suppression

## Data Availability

As the genomic data of this species are from a taonga and thus culturally important species in Aotearoa New Zealand, the data have been deposited in a managed repository that controls access. Raw and analyzed data are available through the Genomics Aotearoa data repository at https://repo.data.nesi.org.nz/, (accessed on accessed on 14 September 2021). This was done to recognise Māori as important partners in science and innovation and as inter-generational guardians of significant natural resources and indigenous knowledge.

## Supplementary Materials

The following are available online at https://www.mdpi.com/article/10.3390/genes12111737/s1. Table S1: This table contains SNP variants detected as outliers in the comparison between the F_0_ and F_1_ cohorts that had genes in proximity. Table S2: This table contains SNP variants detected as outliers in the comparison between the F_1_ and F_2_ cohorts that had genes in proximity. Table S3: This table contains SNP variants detected as outliers in the comparison between the F_1_ and F_2_ cohorts that had genes in proximity. Figure S1: This Figure shows an overview of the methods used to achieve the project goals. Figure S2: This figure shows histograms of *p*-values for runs of LFMM between different comparisons of populations and different values of *K*.
