# Supplementary material for: Genomic Signatures of Domestication Selection in the Australasian Snapper (Chrysophrys auratus)
Source: Genes (Basel). 2021 Oct 29;12(11):1737. doi: 10.3390/genes12111737 (PMC8623400; doi:10.3390/genes12111737)
Supplement: Supplementary file 1 [file genes-12-01737-s001.zip › genes-1400084-supplementary.pdf]

Supplementary Materials

Supplementary Table S1: This table contains SNP variants detected as outliers in the comparison between the F_0 and F_1 cohorts that had genes in proximity. It shows the chromosome the SNP variant was found, its position on the chromosome, the methods that detected it and the name of the gene found in proximity to it.

Chromosome	Bp	Method	Gene name
LG1	21204878	XP-EHH	zgc:66447
LG1	21204878	XP-EHH	slc25a51b
LG5	1026005	XP-EHH	pik3r4
LG5	1027241	XP-EHH	pik3r4
LG5	1026005	XP-EHH	atp2c1
LG5	1027241	XP-EHH	atp2c1
LG8	16240409	XP-EHH	dlx2a
LG8	16246615	XP-EHH	dlx2a
LG8	16240409	XP-EHH	dlx1a
LG8	16246615	XP-EHH	dlx1a
LG8	6330019	XP-EHH	wars2
LG8	6330019	XP-EHH	tbx15
LG20	25449832	XP-EHH	cavin4b
LG20	25449832	XP-EHH	dnah5l
LG20	25449832	XP-EHH	CABZ01038521.1
LG21	12457761	LFMM	CABZ01065423.1
LG21	22721575	LFMM	actr10

Supplementary Table S2: This table contains SNP variants detected as outliers in the comparison between the F_1 and F_2 cohorts that had genes in proximity. It shows the chromosome the SNP variant was found, its position on the chromosome, the methods that detected it and the name of the gene found in proximity to it.

Chromosome	Bp	Method	Gene name
LG21	22721575	LFMM	pgfb
LG22	1816828	XP-EHH	cntnap3
LG24	7259569	LFMM, Bayescan	spry2
LG2	24437692	Bayescan	cast
LG7	9634521	LFMM	mef2b
LG7	9634521	LFMM	tmem161a
LG7	9634521	LFMM	tmem161b
LG8	25251189	LFMM	asic4a
LG8	25252407	LFMM, Bayescan	asic4a
LG9	5256852	LFMM	frmd6
LG10	1227444	LFMM, Bayescan	dock5
LG10	1227444	LFMM, Bayescan	dock1
LG10	3925004	Bayescan	emid1
LG18	20734942	XP-EHH	emid1
LG18	20745147	XP-EHH	emid1
LG10	4477035	LFMM	PITPNM2
LG10	4477035	LFMM	pitpnm3
LG18	25091357	XP-EHH	pitpnm3
LG10	4477035	LFMM	plrdgb
LG18	25091357	XP-EHH	plrdgb
LG11	1610091	LFMM	herc1
LG11	2816662	XP-EHH	herc1
LG11	1610091	LFMM	si:ch211-112g6.4
LG11	1610091	LFMM	dapk2b
LG11	1610091	LFMM	dapk2a
LG11	20299883	LFMM	epha6
LG11	20299883	LFMM	EPHB3
LG11	20299883	LFMM	AL954670.1
LG11	20299883	LFMM	arl6
LG11	20299883	LFMM	ephb2a
LG11	20616751	LFMM	tmco3
LG11	20616751	LFMM	dcun1d2a
LG11	2743034	Bayescan	anxa2a
LG11	2743034	Bayescan	ftr67
LG11	2816662	XP-EHH	znf395a
LG11	2816662	XP-EHH	trpm1a
LG11	35883595	LFMM	adamts17
LG12	11382117	LFMM	p3h4
LG12	11382117	LFMM	nt5c3a
LG12	11382117	LFMM	fkbp10b
LG12	11382117	LFMM	fkbp9
LG12	11382117	LFMM	adam11
LG15	1434167	LFMM	sec31a
LG16	21888792	XP-EHH	fgfr4
LG16	22376561	Bayescan	ik

LG16	22376561	Bayescan	ndufa2
LG16	23333339	LFMM	cadm1b
LG16	23333339	LFMM	cadm1a
LG18	20374531	XP-EHH	qpctla
LG18	20374531	XP-EHH	qpctlb
LG18	20374531	XP-EHH	rrm1
LG18	20374531	XP-EHH	six9
LG18	20734942	XP-EHH	lxn
LG18	20734942	XP-EHH	wdr53
LG18	20745147	XP-EHH	wdr53
LG18	20734942	XP-EHH	srprb
LG18	20745147	XP-EHH	srprb
LG18	20745147	XP-EHH	emilin2a
LG18	21265383	XP-EHH	schip1
LG18	21272263	XP-EHH	schip1
LG18	21289146	XP-EHH	schip1
LG18	21459203	XP-EHH	si:dkey-6n21.13
LG18	21459203	XP-EHH	p2ry1
LG18	21459203	XP-EHH	mfsd1
LG18	23112187	XP-EHH	thap12b
LG18	23112187	XP-EHH	map6b
LG18	23112187	XP-EHH	dyrk1ab
LG18	25445239	XP-EHH	traf4a
LG18	29888560	LFMM	hnrnpl
LG18	29888560	LFMM	irf2bp1
LG18	29888560	LFMM	cd3eap
LG18	29888560	LFMM	irf2bpl
LG18	6852727	LFMM, Bayescan	si:ch73-265h17.1
LG18	6852727	LFMM, Bayescan	si:ch73-265h17.2
**LG19**	**3350723**	**XP-EHH**	**elac2**
**LG19**	**3357023**	**XP-EHH**	**elac2**
**LG19**	**3365779**	**XP-EHH**	**elac2**
**LG19**	**3367929**	**XP-EHH**	**elac2**
**LG19**	**3365779**	**XP-EHH**	**map2k4a**
**LG19**	**3367929**	**XP-EHH**	**map2k4a**
**LG19**	**3365779**	**XP-EHH**	**map2k4b**
**LG19**	**3367929**	**XP-EHH**	**map2k4b**
LG19	4377093	XP-EHH	tbcd
LG19	4377093	XP-EHH	fn3krp
LG19	4377093	XP-EHH	epn3a
LG19	4377093	XP-EHH	epn3b
LG19	4377093	XP-EHH	arl16
LG19	4377093	XP-EHH	znf750
LG21	9596988	XP-EHH	fmn2b
LG21	9596988	XP-EHH	si:ch1073-209e23.1
LG21	9596988	XP-EHH	scg5
LG21	9596988	XP-EHH	grem1a
LG21	9596988	XP-EHH	fmn1
LG21	9596988	XP-EHH	CU695215.3
LG21	9635562	XP-EHH	ryr3

LG21	9703594	XP-EHH	ryr3
LG21	9706219	XP-EHH	ryr3
LG21	9706219	XP-EHH	RYR2
LG22	11438258	XP-EHH	si:ch211-194e18.2
LG22	11438258	XP-EHH	AL831730.1
LG22	11438258	XP-EHH	cfap36
**LG22**	**20343783**	**XP-EHH**	**nsmce4a**
**LG22**	**20356187**	**XP-EHH**	**nsmce4a**
**LG22**	**20343783**	**XP-EHH**	**tmem63bb**
**LG22**	**20343783**	**XP-EHH**	**CU695232.1**
LG22	7176199	XP-EHH	cfap43
LG22	7186310	XP-EHH	cfap43
LG22	7176199	XP-EHH	sfr1
LG22	7186310	XP-EHH	sfr1
**LG22**	**7199122**	**XP-EHH**	**col17a1b**
**LG22**	**7210194**	**XP-EHH**	**col17a1b**
**LG22**	**7215730**	**XP-EHH**	**col17a1b**
**LG22**	**7199122**	**XP-EHH**	**col17a1a**
**LG22**	**7210194**	**XP-EHH**	**col17a1a**
**LG22**	**7199122**	**XP-EHH**	**slka**
**LG22**	**7210194**	**XP-EHH**	**slka**
**LG22**	**7215730**	**XP-EHH**	**slka**
LG22	7210194	XP-EHH	slkb
LG22	7215730	XP-EHH	slkb
LG23	14331052	LFMM	khnyn
LG23	14331052	LFMM	n4bp1
LG23	14331052	LFMM	ripk3
LG23	14331052	LFMM	ripk4
LG23	15167698	LFMM	tnfsf10l

Supplementary Table S3: This table contains SNP variants detected as outliers in the comparison between the F_1 and F_2 cohorts that had genes in proximity. It shows the chromosome the SNP variant was found, its position on the chromosome, the methods that detected it and the name of the gene found in proximity to it.

Chromosome	Bp	Method	Gene name
LG3	10454621	Bayescan	neurl1aa
LG3	27864726	Bayescan	cpe
LG3	27864726	Bayescan	msmo1
LG6	6540643	Bayescan	hcn4l
LG6	6540643	Bayescan	hcn2b
LG6	6540643	Bayescan	CABZ01086574.1
LG11	9916362	Bayescan	mdga1
LG17	21892383	Bayescan	BX324233.1
**LG19**	**3350723**	**XP-EHH**	**elac2**
**LG19**	**3357023**	**XP-EHH**	**elac2**
**LG19**	**3365779**	**XP-EHH**	**elac2**
**LG19**	**3365779**	**XP-EHH**	**map2k4a**
**LG19**	**3365779**	**XP-EHH**	**map2k4b**
LG20	13163319	Bayescan	rnpc3
LG21	11657718	Bayescan	ext1a
LG21	11657718	Bayescan	ext1b
LG21	16908061	Bayescan	nhsl1b
**LG22**	**20343783**	**XP-EHH**	**nsmce4a**
**LG22**	**20343783**	**XP-EHH**	**tmem63bb**
**LG22**	**20343783**	**XP-EHH**	**CU695232.1**
LG22	26162226	Bayescan	zmiz1a
**LG22**	**7199122**	**XP-EHH**	**col17a1b**
**LG22**	**7210194**	**XP-EHH**	**col17a1b**
**LG22**	**7199122**	**XP-EHH**	**col17a1a**
**LG22**	**7210194**	**XP-EHH**	**col17a1a**
**LG22**	**7199122**	**XP-EHH**	**slka**
**LG22**	**7210194**	**XP-EHH**	**slka**
**LG22**	**7210194**	**XP-EHH**	**slkb**

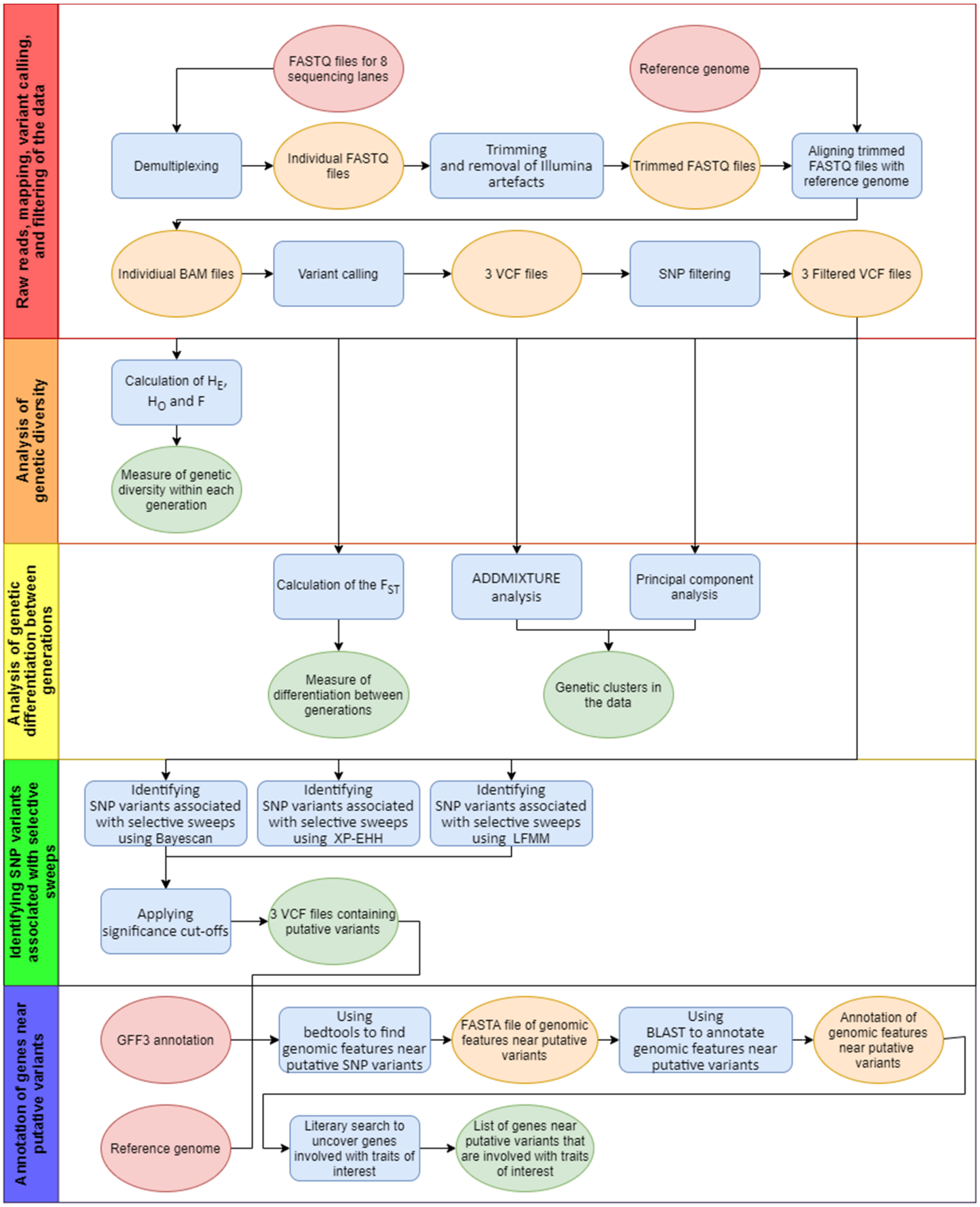

Supplementary Figure S1: This Figure shows an overview of the methods used to achieve the project goals. Red circles indicate input data, orange circles indicate intermediate data and green circles indicate a final result that is serves to help fulfil one of the project goals. Finally, blue squares indicate an action or analysis that was performed.

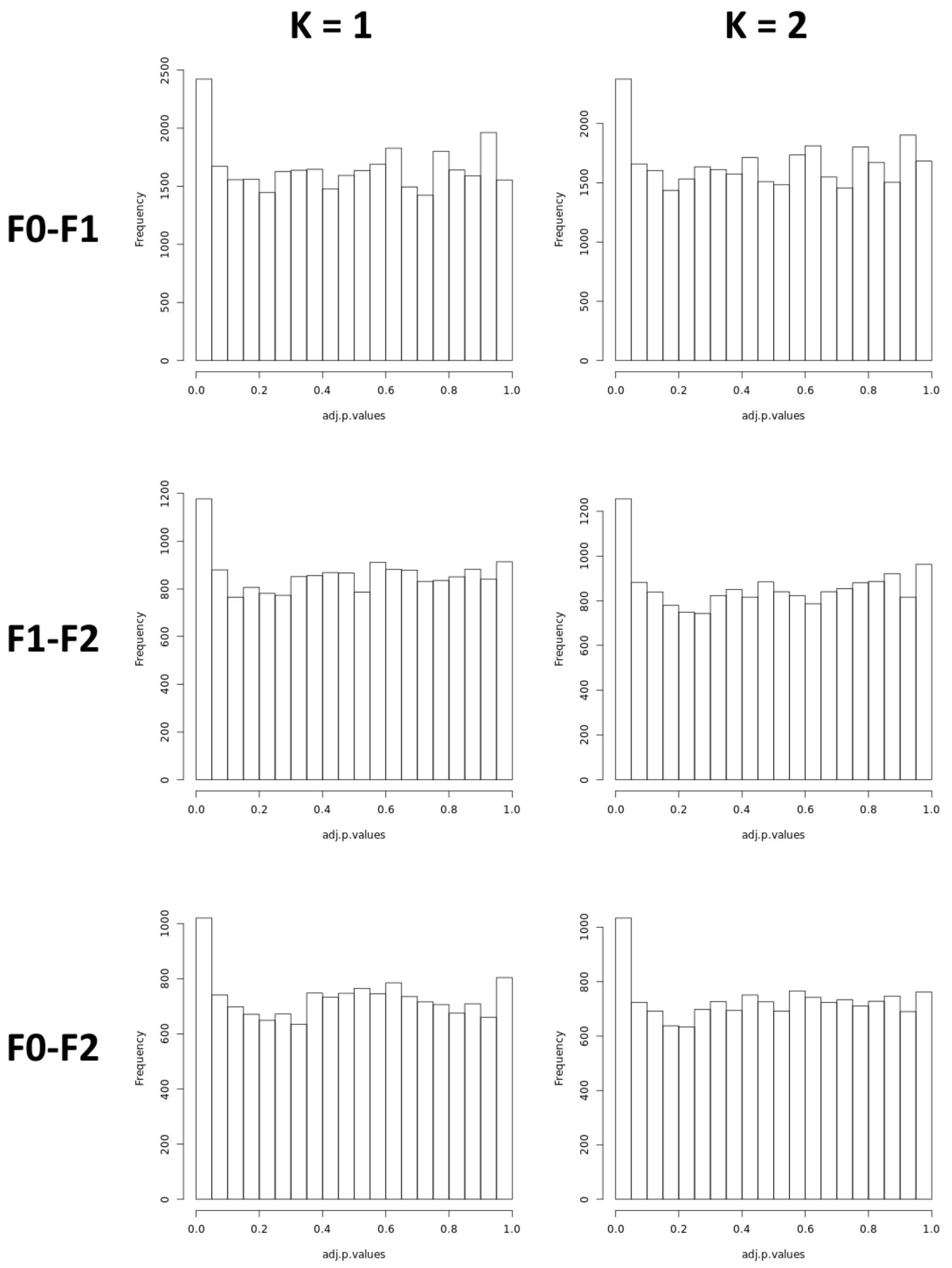

Supplementary Figure S2: This figure shows histograms of p-values for runs of LFMM between different comparisons of populations and different values of K. The pattern associated with properly calibrated results is a mostly flat graph, with a peak near 0.0. As can be seen in the graphs, this can be seen both with K = 1 and K = 2. With no Large difference between the two, the value of K = 2 was chosen because of the two cohorts forming the original F_0.
